# Mineral vs. Organic Amendments: Microbial Community Structure, Activity and Abundance of Agriculturally Relevant Microbes Are Driven by Long-Term Fertilization Strategies

**DOI:** 10.3389/fmicb.2016.01446

**Published:** 2016-09-14

**Authors:** Davide Francioli, Elke Schulz, Guillaume Lentendu, Tesfaye Wubet, François Buscot, Thomas Reitz

**Affiliations:** ^1^Department of Soil Ecology, Helmholtz Centre for Environmental Research – UFZHalle, Germany; ^2^Department of Ecology, University of KaiserslauternKaiserslautern, Germany; ^3^German Centre for Integrative Biodiversity Research (iDiv), Halle-Jena-LeipzigLeipzig, Germany

**Keywords:** long-term fertilization, soil nutrients, microbial biomass, microbial activity, 454 pyrosequencing, soil microbial communities

## Abstract

Soil management is fundamental to all agricultural systems and fertilization practices have contributed substantially to the impressive increases in food production. Despite the pivotal role of soil microorganisms in agro-ecosystems, we still have a limited understanding of the complex response of the soil microbiota to organic and mineral fertilization in the very long-term. Here, we report the effects of different fertilization regimes (mineral, organic and combined mineral and organic fertilization), carried out for more than a century, on the structure and activity of the soil microbiome. Organic matter content, nutrient concentrations, and microbial biomass carbon were significantly increased by mineral, and even more strongly by organic fertilization. Pyrosequencing revealed significant differences between the structures of bacterial and fungal soil communities associated to each fertilization regime. Organic fertilization increased bacterial diversity, and stimulated microbial groups (Firmicutes, Proteobacteria, and Zygomycota) that are known to prefer nutrient-rich environments, and that are involved in the degradation of complex organic compounds. In contrast, soils not receiving manure harbored distinct microbial communities enriched in oligotrophic organisms adapted to nutrient-limited environments, as Acidobacteria. The fertilization regime also affected the relative abundances of plant beneficial and detrimental microbial taxa, which may influence productivity and stability of the agroecosystem. As expected, the activity of microbial exoenzymes involved in carbon, nitrogen, and phosphorous mineralization were enhanced by both types of fertilization. However, in contrast to comparable studies, the highest chitinase and phosphatase activities were observed in the solely mineral fertilized soil. Interestingly, these two enzymes showed also a particular high biomass-specific activities and a strong negative relation with soil pH. As many soil parameters are known to change slowly, the particularity of unchanged fertilization treatments since 1902 allows a profound assessment of linkages between management and abiotic as well as biotic soil parameters. Our study revealed that pH and TOC were the majors, while nitrogen and phosphorous pools were minors, drivers for structure and activity of the soil microbial community. Due to the long-term treatments studied, our findings likely represent permanent and stable, rather than transient, responses of soil microbial communities to fertilization.

## Introduction

Global demand for agricultural crops is increasing and global food production is already dependent on intensive agricultural management. Fertilization is a common farming practice, in which organic and inorganic fertilizers are used primarily to improve plant nutrition and hence crop productivity. The type and quantity of fertilizer amendment affects not only crop yields but also the physico-chemical properties of the soil, which, in the long-term, have a significant influence on soil fertility and productive capacity (Saha et al., 2008). Fertilization with organic amendments typically improves soil fertility and structure by increasing soil nutrient status and organic matter content (Reganold et al., 1987; Maeder et al., 2002; Liang et al., 2012). Consequently organic fertilization enhances soil microbial biomass (Esperschutz et al., 2007; Gomiero et al., 2011) and activity (Ros et al., 2003). Mineral fertilizers, especially nitrogen (N) inputs, have made a major contribution to the impressive increases in crop yield achieved since the 1950s (Robertson and Vitousek, 2009). Despite the positive effects of inorganic fertilizers on crop yields, there can be indirect negative effects on soil quality arising from the complex transformations of N in the soil. The application of ammonium fertilizers may reduce soil pH by causing a high rate of proton release to the soil due to enhanced nitrification processes and ammonium uptake by the plants. As a consequence, soil acidification can lead to deficiency of many nutrients, decreases in crop yield and deterioration in soil fertility (Barak et al., 1997). Concerning the effects of mineral fertilizers on soil microbial growth and activity, contrasting results have been found so far. Recently, Geisseler and Scow (2014) examined 107 datasets of 64 long-term trials from around the world, reporting that in most of the studies mineral fertilization led to a significant increase in the soil microbial biomass, while other field studies based on short-term application of N amendments found opposite results (Lupwayi et al., 2011; Roberts et al., 2011; Lazcano et al., 2013). In addition, positive and negative effects (i.e., both increases and decreases in soil microbial activities) on microbial soil enzyme activities have been reported in soil receiving inorganic fertilizers (Gianfreda and Ruggiero, 2006; Guo et al., 2011; Nannipieri et al., 2012). By comparing the impacts of mineral and organic fertilization on soil communities, higher soil microbial biomass and different community structures have already been observed in agricultural soil with regular organic manure application (Marschner et al., 2004; Esperschutz et al., 2007; Lentendu et al., 2014). Although several studies were addressed to survey the effects of fertilization on the soil ecosystem, most of them focused on short-term responses, which are expected to differ considerably from those in the long-term (Liang et al., 2015; Eo and Park, 2016). Long-term fertilization can have more persistent impacts on soil characteristics (Rousk et al., 2011; Körschens et al., 2013), plant growth (Clark et al., 2007), and microbial diversity and activity (Giacometti et al., 2014; Hartmann et al., 2015). For instance, the effects of inorganic fertilizers can differ according to its application period, and it may take a long time for a soil ecosystem to reach an equilibrium state (Fauci and Dick, 1994; Moscatelli et al., 2008). Thus, many previous studies have emphasized the importance of long-term field experiments to evaluate the effects of different farming systems on soil quality and productivity (Rasmussen et al., 1998; Marschner et al., 2003; Chakraborty et al., 2011). The “Static Fertilization Experiment” (Bad Lauchstädt, Saxony-Anhalt, Germany), established in 1902, is one of the oldest agricultural field experiments worldwide, and it aims to provide a comprehensive understanding of the effects of long-term fertilization on the yields and quality of crops as well as on soil fertility and ecosystem functions (Merbach and Schulz, 2012). In this study, we used a 454-pyrosequencing approach of the bacterial 16S rDNA and the fungal ITS region to examine the response of soil microbial communities to 113 years of different fertilization regimes (mineral, organic, and combined fertilization) in the “Static Fertilization Experiment.” Moreover, we analyzed the influence of long-term nutrient addition on soil properties, microbial biomass and on the activity of soil hydrolases involved in carbon, nitrogen and phosphorus cycles in soil.

The overall objective of the present study was to use a multidisciplinary approach to carry out an in-depth survey on the long-term effects of different fertilization strategies on the activity and composition of soil microbiome. Assuming that each fertilization treatment has a different influence on soil organic matter and nutrient levels, we hypothesized that (i) the soil edaphic properties, which are strongly affected by long-term fertilization, in turn affect the structure, quantity and diversity of soil microbial communities; and (ii) such variations in microbial communities would reflect changes in microbial activity and function. Moreover, we hypothesized that (iii) the community changes caused by long-term fertilization included shifts in the abundance of various plant-beneficial and detrimental soil microorganisms, thus influencing agro-ecosystem performance and stability.

## Materials and Methods

### Study Site, Experimental Design, and Soil Sampling Strategy

The Bad Lauchstädt research station is located in the Hercynian dry region of central Germany (11°53′E, 51°24′N), which is characterized by a mean annual precipitation of 484 mm and a mean annual temperature of 8.7°C. The “Static Fertilization Experiment,” situated on a loess-derived loam soil (Haplic Chernozem; FAO) consisting of 21.0% clay, 67.8% silt and 11.2% sand, was established in 1902 and consists of 18 different fertilization treatments based on a 4-year crop rotation. A comprehensive description of the experimental site and treatments was given by Blair et al. (2006). The crop rotation consists of sugar beet (*B. vulgaris*), spring barley (*H. vulgare*), potato (*S. tuberosum*) and winter wheat (*T. aestivum*). Each year, all crops are grown in parallel on the four crop strips at the experimental site. The fertilization treatments chosen for this study were no fertilization (NF), mineral fertilization (NPK), farmyard manure fertilization (FYM), and combined farmyard manure and mineral fertilization (FYM+NPK). Soils fertilized with FYM received a total of 20 t ha^-1^ of farmyard manure (solid, cattle manure with bedding) in every other year while the soils fertilized with NPK received an annual dose of mineral fertilizer (calcium ammonium nitrate + superphosphate + potassium chloride, Supplementary Table S1). The application of fertilizers is carried out in November after the crop growing season. Details on crop yield (for 2012; see Supplementary Table S2) and agricultural management practices are described elsewhere (Blair et al., 2006).

Soil samples were collected in October 2012 before fertilizers had been applied and crops had been sown. Sampling was performed for the four selected fertilization treatments on all four crop strips. The size of each plot is 265 m^2^, but no independent replicates were included when the long-term fertilization experiment was established. We therefore used an adapted sampling design in order to obtain five soil pseudo-replicates representative of each of the four fertilization treatments (Supplementary Figure S1). Each of the four plots representing one fertilization treatment was divided into five subplots. From each subplot, 50 randomly distributed soil cores (up to a depth of 20 cm and 1.2 cm in diameter) were taken with a sampling probe and pooled. Afterward we combined the corresponding soil samples from each strip, obtaining five composite soil replicates (A–E; Supplementary Figure S1) for each fertilization treatment. Soil samples were sieved through a 2 mm mesh at the sampling site and kept at 4°C for chemical and microbial enzyme analyses and at -80°C for molecular analyses.

### Analytical Methods

#### Total Carbon and Nitrogen

Total organic carbon (TOC) and total nitrogen (TN) contents of the soil samples were determined in triplicate by dry combustion using a Vario EL III C/H/N analyser (Elementar, Hanau, Germany). Since the carbonate concentration of the soils was negligible (<2%), the total C concentration measured was considered to represent TOC.

#### Hot Water Extractable C

Hot water extraction from air-dried soil samples was performed in order to quantify the labile organic C pool, i.e., the potentially mineralizable and decomposable fraction of the TOC. This was done by boiling a soil/water suspension (1:5, w/v) for 1 h under reflux, according to Schulz (2002). After cooling to room temperature, 0.1 ml of 1 M MgSO_4_ was added to facilitate soil sedimentation. The sedimented suspensions were then centrifuged for 10 min at 6700 × *g* to obtain clear extracts. All water extracts were filtered (0.45 μm Minisart RC25 single-use syringe membrane filters, PP-housing, Sartorius AG, Göttingen, Germany) prior to the determination of hot water extractable C (HWC) concentrations (mg kg^-1^), which was done using an elemental analyser for liquid samples (Multi N/C, Analytik Jena, Germany).

#### Mineral N Analysis

NH_4_^+^-N and NO^-^_3_-N were extracted from 10 g of fresh soil with 1 M KCl (1:4 w/v) by shaking horizontally for 1.5 h. After filtration of the suspension (Whatman Schleicher and Schuell 595 1/5 Ø 270 mm), the concentrations of NH_4_^+^-N and NO_3_^-^-N in the clear extracts were measured using a flow injection analyzer (FIAstar 5000, Foss GmbH, Rellingen, Germany).

#### Plant Available P Analysis

Plant available P was extracted from fresh soil with double lactate (1:50 w/v, pH 3.6, 1.5 h; Riehm, 1943). After filtration of the suspension (Whatman Schleicher and Schuell 595 1/5 Ø 270 mm), the extracted P was quantified colorimetrically using the molybdenum blue method (Murphy and Riley, 1962).

#### Microbial Biomass Carbon Using Substrate Induced Respiration

Microbial biomass carbon (MBC) was estimated with 20 g dry equivalent of field-moist soil according to Anderson and Domsch (1978). All samples were pre-incubated at 22°C for10 days in polyethylene vessels. The vessels were then incubated in an automatic respirometer (Respicond V, Nordgren Innovations AB, Sweden) at a constant temperature of 22°C, and measurements of CO_2_ evolution were taken hourly. After 24 h samples were amended with 0.8 g of a glucose/talcum mixture (1:1.5 w/w). From the initial (4–6 h) CO_2_ response, we calculated the SIR-MBC using the regression equation of Anderson and Domsch (1978). CO_2_ rate from the respirometer readings, given as mg CO_2_ g^-1^h^-1^, was converted into μl CO_2_ g^-1^h^-1^.

### Soil Enzyme Assays

Determination of the activities of five hydrolytic enzymes was based on 4 methylumbelliferone (MUB)-coupled substrates (German et al., 2011). 4-MUB-β-D-cellobioside, 4-MUB-β-D-glucoside, 4-MUB-β-D-xyloside, 4-MUB-*N*-acetyl-β-D-glucosaminide and 4-MUB-phosphate were used to estimate the activities of enzymes involved in carbon (β-glucosidase, cellobiohydrolase, xylosidase), nitrogen (*N*-acetylglucosaminidase), and phosphorus (phosphatase) acquisition, respectively (Supplementary Table S3). The substrate concentrations in the assays were optimized in a pre-test to ensure that each enzyme was assayed under saturating conditions, in order to avoid an underestimation of enzyme activities (Nannipieri and Gianfreda, 1998). Soil suspensions were prepared by adding 0.5 g of fresh soil to 50 ml of 50 mM Tris (pH 6.4, according to the mean pH of all soil samples) and subsequent sonication for 5 min. MUB standards (0.16, 0.625, 1.25, and 2.5 μM) dissolved in buffer and soil suspensions were used to calculate the emission and quench coefficients for each sample. Enzyme activities were calculated according to German et al. (2011). We additionally determined biomass-specific enzyme activities (μmol g C^-1^h^-1^) by dividing enzyme activities (nmol g soil^-1^ h^-1^) by microbial biomass (μg C g soil^-1^).

### DNA Extraction, Amplicon Library Preparation, and Pyrosequencing

Total soil DNA was extracted from 0.25 g of soil using a ZR Soil Microbe DNA MiniPrep kit (Zymo Research, Irvine, CA, USA), according to the manufacturer’s instructions. DNA concentrations in the extracts were quantified using a NanoDrop ND-1000 spectrophotometer (NanoDrop Technologies, Wilmington, DE, USA). Bacterial and fungal amplicon libraries were produced using custom fusion primers. We used the primer pair BAC 341F and BAC 907R to amplify the V3–V5 region of the bacterial 16S rRNA gene (Muyzer and Smalla, 1998). We used the primer pair ITS1F and ITS4 to amplify the fungal internal transcribed spacer (ITS) rRNA region. Custom primers for unidirectional sequencing were constructed using 10 bp barcodes, sequencing primers and the BAC 907R and ITS4 primers (Lentendu et al., 2014); PCR amplifications were performed in triplicate with a total volume of 50 μL reaction mix containing 1 μL of soil DNA template, 25 μL GoTaq Green Master Mix (Promega, Mannheim, Germany) and 1 μL of each primer (25 μM).

The thermal profile used for preparation of bacterial rDNA amplicon libraries was as follows: initial denaturation at 98°C for 1 min, 30 cycles of denaturation at 95°C for 45 s, annealing at 57°C for 45 s, and extension at 72°C for 90 s, followed by a final extension period at 72°C for 10 min. The reactions for preparation of fungal ITS rDNA amplicon libraries were performed using touchdown PCR conditions with an initial denaturation for 5 min at 95°C followed by: (1) 10 cycles of 94°C for 30 s, 60–50°C (-1°C per cycle) for 45 s and 72°C for 2 min; and (2) 30 cycles of 94°C for 30 s, 50°C for 45 s and 72°C for 2 min, with a final extension step of 10 min. The PCR products were analyzed using a 1.5% agarose gel, and amplicons from the triplicate PCRs were then pooled and purified using a Qiagen Gel Extraction kit (Qiagen, Hilden, Germany). The amount of DNA in each of the purified samples was measured using the PicoGreen dsDNA assay (Invitrogen, Carlsbad, CA, USA). Samples were pooled at equimolar concentration and sequenced unidirectionally using a 454 Titanium amplicon sequencing kit and a Genome Sequencer FLX 454 System (454 Life Sciences/Roche Applied Biosystems, Mannheim, Germany) at the Department of Soil Ecology, Helmholtz Centre for Environmental Research (UFZ, Halle, Germany).

### Pyrosequencing Data Analysis

Raw reads were first demultiplexed and they were further quality-trimmed if they carried the expected barcode and forward primer sequences with maxima of one and four mismatches, respectively, using the MOTHUR software package (Schloss et al., 2009). For bacterial 16S reads, sequences with a minimum average quality of a Phred score of 30, a minimum length of 500 nt, a maximum homopolymer length of eight nucleotides, and no ambiguous nucleotides, were selected as high quality reads. Similarly, fungal ITS reads were retained as high quality reads if they had a minimum average quality of a Phred score of 25, a minimum length of 450 nt, a maximum homopolymer length of eight nucleotides and a maximum of eight ambiguous nucleotides. High quality 16S reads were first aligned against the aligned version of the reference Silva SSU database (Release 115, non-redundant, clustered at 99% similarity; Quast et al., 2013). Chimeras were further removed from the aligned 16S reads and the high quality ITS reads using UCHIME (Edgar et al., 2011) as implemented in MOTHUR. Unique sequences were sorted by decreasing abundance for both the 16S and the ITS dataset and were clustered into OTUs using CD-HIT-EST (Fu et al., 2012) at a threshold of 97% pairwise similarity. Low abundant OTUs with 3 or fewer reads were removed as they potentially originated from sequencing artifacts (Kunin et al., 2010). Bacterial 16S OTU representative sequences were classified against the non-aligned version of the above mentioned Silva database using the MOTHUR implementation of the Wang et al. (2007) classifier. Representative sequences for fungal ITS OTUs were classified against the dynamic version of the UNITE database (version 6, January’ 14; Kõljalg et al., 2013). Those ITS sequences which could not be assigned further than the kingdom Fungi were reclassified against the previous database augmented with all eukaryotic and non-fungal ITS sequences retrieved from GenBank (release 202, Benson et al., 2008), in order to remove non-target organism sequences (those from OTUs not affiliated to fungi). If a sequence could still not be assigned to a fungal phylum, it was classified against the full version of the UNITE database in order to improve its taxonomic annotation.

### Nucleotide Accession Number

The 454 pyrosequencing data generated for this study were submitted to the European Nucleotide Archive (ENA) under accession numbers PRJEB9307 and PRJEB9305.

### Statistical Analysis

Univariate Analysis of Variance (ANOVA) followed by Tukey’s honestly significant difference (HSD) *post hoc* test was used to test for differences in soil chemical properties, microbial biomass, and enzyme activities among the four treatments investigated in this work. All the variables were tested for normality using Shapiro–Wilk and Jarque–Bera tests and the equality of group variances was examined using Levene’s test. A log_10_ transformation [log_10_ (*x* + 1)] was applied to all variables that did not meet the parametric assumptions. Correlation among the soil parameters and between soil parameters and enzyme activities were determined using Spearman’s rank correlation. Diversity of the bacterial and fungal community was assessed by calculating the Shannon–Wiener index. Differences in bacterial and fungal OTU richness and diversity were compared using an ANOVA followed by a Tukey’s HSD *post hoc* test. Similarities in the bacterial and fungal community structure among the four fertilization treatments were investigated using an analysis of the similarity (ANOSIM) algorithm. Non-metric multidimensional scaling (NMDS) based on the Bray–Curtis dissimilarity index was used to visualize the patterns of distribution of microbial communities in relation to fertilization treatments. Permutational multivariate analysis of variances (PERMANOVA) based on the Bray–Curtis dissimilarity index was performed to analyze the effect of fertilization treatment on community composition in each data set, using 999 permutations for each test. We used the option “strata” in the R package “vegan” to constrain the permutation of samples within each fertilization group. A model of multivariate analysis of variance was constructed using distance-based redundancy analysis (dbRDA) based on the Bray–Curtis distance to determine the environmental variables that were most influential on the bacterial and fungal community compositions. Marginal tests were performed to determine the amounts of variation explained by the selected variables. Significance tests were performed through nonparametric permutation, which does not rely on the assumption of multivariate normality (Taylor et al., 2009). All the data were analyzed with R version 2.15.3 (R Core Team, 2012).

## Results

### Soil Chemical Properties and Microbial Biomass Carbon

The soil parameters have been strongly influenced by the long-term application of mineral and organic fertilizers (**Table 1**). The unfertilized soil (NF) had a pH of 6.85, while the soil pH had decreased to a value of 5.74 in the NPK treated soil. Slightly lower pH values, compared to the NF soil, of 6.56 and 6.42 were also observed in the organic (FYM) and combined (FYM+NPK) fertilized soils, respectively. Long-term mineral and organic nutrient inputs induced a significant increase (*p* < 0.05) in TOC, HWC, TN, and available phosphorus [P_(DL)_] in soil and they increased in the order NF < NPK < FYM < FYM+NPK, whereas an opposite trend was observed for C/N ratio. TOC was positively correlated with TN (*r* = 0.97, *p* < 0.01), HWC (*r* = 0.88, *p* < 0.01) and P_(DL)_ (*r* = 0.90, *p* < 0.01). Furthermore, HWC was positively correlated with TN (*r* = 0.94, *p* < 0.01) and P_(DL)_ (*r* = 0.95, *p* < 0.01) and the latter was also strongly correlated with TN (*r* = 0.93, *p* < 0.01). No significant differences in soil ammonium concentration were observed among the soils studied, with the exception of the one fertilized with NPK, which showed a higher ammonium content. Nitrate concentrations were significantly higher (*p* < 0.05) in all fertilized soils and increased in the order NF < FYM < NPK < FYM+NPK.

**Table 1:** Chemical and biological properties of soil samples.

	NF	NPK	FYM	FYM+NPK
pH	6.85 (0.04)^a^	5.74 (0.05)^c^	6.56 (0.03)^b^	6.42 (0.05)^b^
TOC (g/kg)	160 (1.2)^d^	188 (4.1)^c^	210 (3.7)^b^	229 (2.2)^a^
TN (g/kg)	11 (0.2)^d^	14 (0.4)^c^	17 (0.8)^b^	20 (0.2)^a^
HWC (mg/kg)	284 (0.62)^d^	360 (14.3)^c^	451 (5.9)^b^	518 (5.8)^a^
NH_4_^+^-N (mg/kg)	0.76 (0.03)^b^	1.30 (0.04)^a^	0.72 (0.05)^b^	0.92 (0.04)^b^
NO_3_^-^-N (mg/kg)	10.1 (0.1)^d^	17.9 (0.2)^b^	13.71 (0.3)^c^	28.1 (0.3)^a^
P_(DL)_ (mg/kg)	44.6 (2.2)^d^	83.0 (1.4)^c^	124.7 (1.4)^b^	171.6 (2.3)^a^
C/N (ratio)	14.1 (0.05)^a^	13.9 (0.3)^a^	12.4 (0.4)^b^	11.3 (0.05)^b^
MBC (μg/g)	131 (7.9)^c^	146 (7.7)^c^	197 (10.9)^b^	239 (13.7)^a^

*Values are means and letters denote significant differences among the treatments (p < 0.05). Standard error of the means are indicated in parentheses. NF, no fertilization; NPK, mineral fertilization; FYM, organic fertilization; FYM+NPK, organic and mineral fertilization; TOC, total organic carbon; TN, total nitrogen; HWC, hot water extractable carbon; P_(DL)_, plant available phosphorous; MBC, microbial biomass carbon.*

Farmyard manure fertilized soils showed a strong increase of MBC, while long-term application of mineral fertilizer induced only a slight increase in MBC relative to the unfertilized treatments (**Table 1**). MBC increased by 10, 53, and 79% in the NPK, FYM, and FYM+NPK fertilized soils, respectively, compared to NF soil. A significant positive correlation (*p* < 0.01) was found between MBC and the amount of TOC, TN, and HWC with r values of 0.71, 0.73, and 0.65, respectively.

### Enzyme Activities

**FIGURE 1:**
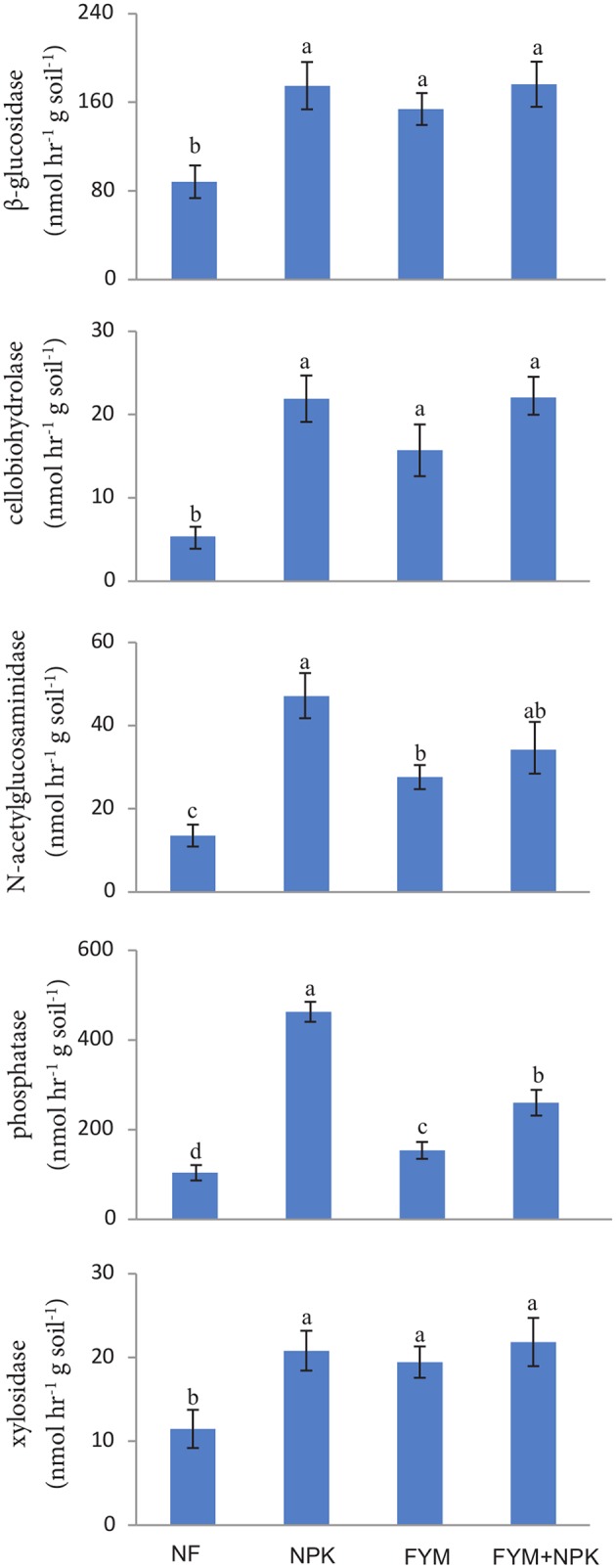
**Enzymatic activities measured in the four soils studied.** Different letters indicate significant differences based on Tukey’s HSD test *p* < 0.05. Error bars represent one standard error of the mean (*n* = 5).

Long-term fertilization significantly increased (*p* < 0.05) the activities of β-glucosidase, cellobiohydrolase, *N*-acetylgluco saminidase, phosphatases, and xylosidase compared to their activities in the NF soil (**Figure 1**). β-glucosidase and xylosidase activities were approximately two times higher in all fertilized treatments than in the NF soil. Cellobiohydrolase activity was about four times higher in the NPK and FYM+NPK soils, and three times higher in the FYM soil, than in the NF soil. *N*-acetylglucosaminidase showed the highest activity in the NPK soil, followed by FYM+NPK and FYM soils, while in the NF soil its activity was low. The phosphatase activity in the NPK soil was nearly five times higher than that in the control, three times higher than that in the FYM soil and almost two times higher than that in the FYM+NPK treatment. The hydrolase activities were significantly correlated among each other and β-glucosidase, cellobiohydrolase and xylosidase were further positively correlated with TOC (*r* = 0.59; *r* = 0.61; *r* = 0.54; respectively, *p* < 0.05) and with TN (*r* = 0.53; *r* = 0.59; *r* = 0.47; respectively, *p* < 0.05). *N*-acetylglucosaminidase and phosphatase activities were negatively related to pH (*r* = -0.83; *r* = -0.91, respectively, *p* < 0.001) and positively correlated with ammonium (*r* = 0.57; *r* = 0.60, respectively, *p* < 0.01) and nitrate (*r* = 0.65; *r* = 0.69, respectively, *p* < 0.01) concentrations.

### Bacterial and Fungal Richness and Diversity

A total of 200,477 16S bacterial and 76,267 ITS fungal raw reads were obtained from the 20 soil samples (4 treatments × 5 replicates). After quality filtering steps and exclusion of singletons doubletons and tripletons, 135,761 bacterial and 54,560 fungal high quality reads were recovered. Sequences were clustered into 3,122 bacterial and 610 fungal OTUs.

**FIGURE 2:**
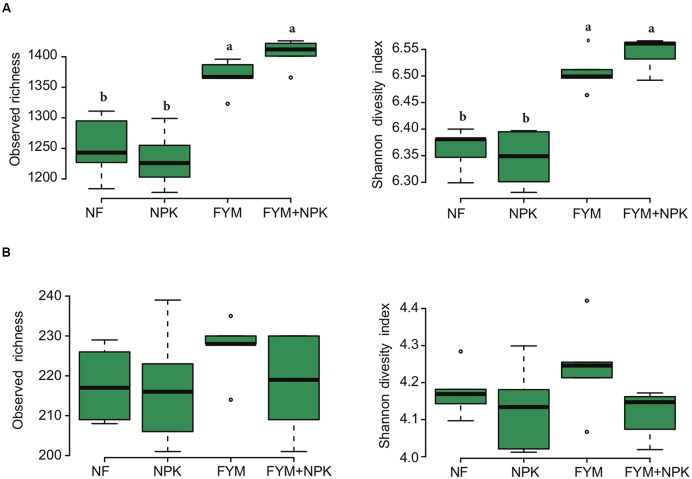
**Box plots of the observed richness and Shannon’s diversity index of (A) bacterial and (B) fungal community in the four soils studied.** Different letters indicate significant differences based on Tukey’s HSD test *p* < 0.05.

The bacterial and fungal rarefaction curves of the observed OTUs did not yet reach a plateau (Supplementary Figure S2) but the observed richness covered between 69–74% and 70–85% of the estimated total bacterial and fungal richness, respectively (Supplementary Table S4). Bacterial diversity and richness estimators were significantly higher (*p* < 0.05) in soil fertilized with farmyard manure (FYM and FYM+NPK) compared to the NF and NPK soils, while no significant differences were observed for fungal diversity (*p* = 0.22, Shannon index) and richness (*p* = 0.49, observed richness) among the four treatments (**Figure 2**). Overall, bacterial sequences were affiliated to 27 phyla (Supplementary Figure S3), 49 classes, 108 orders, 203 families and 331 genera. Actinobacteria were the most abundant phylum, comprising approximately 40.6% of the reads across all samples (793 OTUs), followed by Proteobacteria (25% of reads; 897 OTUs), Acidobacteria (10.8% of reads; 372 OTUs), Chloroflexi (10.1%, of reads; 310 OTUs) and Firmicutes (7.3% of reads; 387 OTUs). A small proportion of members of the Gemmatimonadetes (2.9% of reads; 139 OTUs), Nitrospirae (1.4% of reads; 28 OTUs) and Bacteroidetes (1.2% of reads; 149 OTUs) was also detected. Within the Proteobacteria, subdivisions α, β, δ, and γ accounted for 13.5, 4.2, 3.5, and 3.7% of the total bacterial reads, respectively. Members of the phylum Actinobacteria, such as *Nocardioides* (6.7%), *Gaiella* (5.5%), *Arthrobacter* (4.6%), *Blastococcus* (4.4%) and *Streptomycetes* (3.1%), were the most abundant bacterial genera detected across all soil samples.

Overall, fungal sequences were associated with five phyla (Supplementary Figure S4), 16 classes, 43 orders, 68 families, and 131 genera. Taxonomic assignment of the fungal OTUs showed that Ascomycota (76.4% of reads; 431 OTUs) was the dominant phylum, followed by Basidiomycota (12.6% of reads; 111 OTUs), Zygomycota (10.4% of reads; 30 OTUs), Chytridimycota (0.3% of reads; 14 OTUs), and Glomeromycota (0.02% of reads; 2 OTUs). At the class level, Sordariomycetes (51% of the total fungal reads), Dothideomycetes (14%), Eurotiomycetes (5%) and Leotiomycetes (2%) were identified as the main contributors to relative abundance within the Ascomycota, while Agaricomycetes (3%) and Tremellomycetes (7%) were the most abundant classes of Basidiomycota. Among the Ascomycota, filamentous fungi like *Verticillium*, *Alternari*a, *Fusarium*, and *Podospora* were the most abundant genera, accounting for 13.2, 3.9, 4.5, and 4.1%, respectively, of the total fungal reads. *Cryptococcus* (3.6%) was the most abundant genus of Basidiomycota. Over 10% of the fungal sequences were assigned to the genus *Mortierella*, which was the only genus recognized within the phylum Zygomycota.

### Differences in Bacterial Community Structures

**FIGURE 3:**
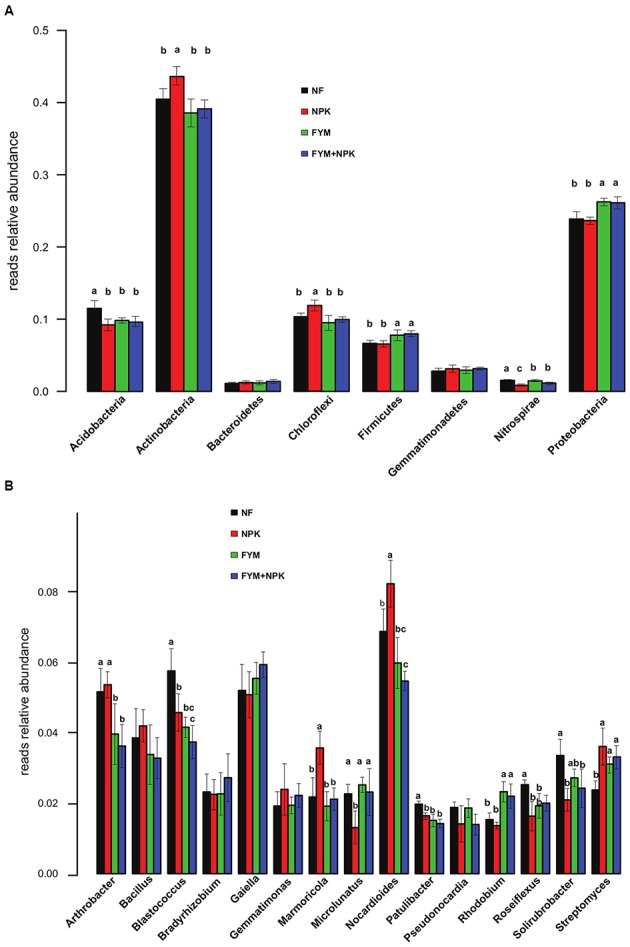
**Relative abundances of the major bacterial phyla (A) and genera (B) in the four soils studied.** Different letters indicate significant differences based on Tukey’s HSD test *p* < 0.05. Error bars represent standard deviation (*n* = 5).

The relative abundance of the dominant bacterial taxa varied markedly among the four soils studied (**Figure 3A**). Overall, the proportion of Actinobacteria and Chloroflexi was significantly higher in the NPK soil than in the unfertilized and organic-fertilized soils (*p* < 0.05). In contrast, Proteobacteria and Firmicutes were relatively more abundant in farmyard manure-fertilized soils (FYM and FYM+NPK, *p* < 0.05), while the NF soil showed a significantly (*p* < 0.05) higher proportion of Acidobacteria and Nitrospirae. Within the phylum Proteobacteria, the relative abundances of Alphaproteobacteria and Gammaproteobacteria were significantly higher in the FYM-amended soils than in NF and NPK soils (*p* < 0.05; data not shown). At the genus level, shifts in the 15 most abundant bacterial genera were also detected among the treatments (**Figure 3B**). OTUs assigned to *Nocardioides* and *Marmicola* were relatively more abundant in the NPK-treated soil (*p* < 0.05), while *Rhodobium* was the only genus with a significant increase (*p* < 0.05) in relative abundance in the FYM-amended soils. Long-term fertilization induced a significant reduction (*p* < 0.05) in the relative abundance of the genera *Blastococcus*, *Patulibacter*, and *Roseiflexus* compared to the untreated soil, while at the same time causing an increase in the relative abundance of the genus *Streptomyces*. FYM fertilization also resulted in a significant decrease (*p* < 0.05) in the relative abundance of the genus *Arthrobacter*.

**FIGURE 4:**
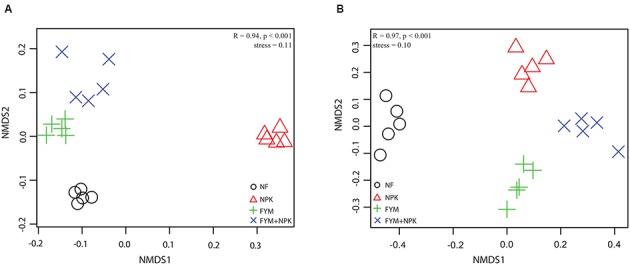
**Non-metric multidimensional scaling (NMDS) ordination of (A) bacterial and (B) fungal community in the four soils studied.** Bonferroni-corrected *p*-values were applied in all cases when more than two groups were compared with ANOSIM. (R = degree of separation between test groups ranging from -1 to 1; *R* = 0, not different; *R* = 1, completely different; *p*-values were based on 9999 permutations).

Analysis of the similarity revealed a significant variation in bacterial community composition among the four fertilization treatments (*r* = 0.941, *p* < 0.001), and differences in community structure were highlighted in the NMDS ordination (**Figure 4**). PERMANOVA confirmed the effect of fertilization on bacterial community structure (**Table 2**): organic fertilization and mineral fertilization explained, respectively, 15.3 and 14.4% of the dissimilarities in community composition (*p* = 0.001 in both cases).

**Table 2:** Permutational multivariate analysis of variance exploring the differences in community composition (from Bray–Curtis dissimilarity matrices) in each data set separately for the different fertilization treatments.

	Bacteria		Fungi	
Tested parameters	*R*^2^	*p*-value	*R*^2^	*p*-value
FYM	0.144	0.001	0.194	0.001
NPK	0.153	0.001	0.228	0.001

### Differences in Fungal Community Structure

**FIGURE 5:**
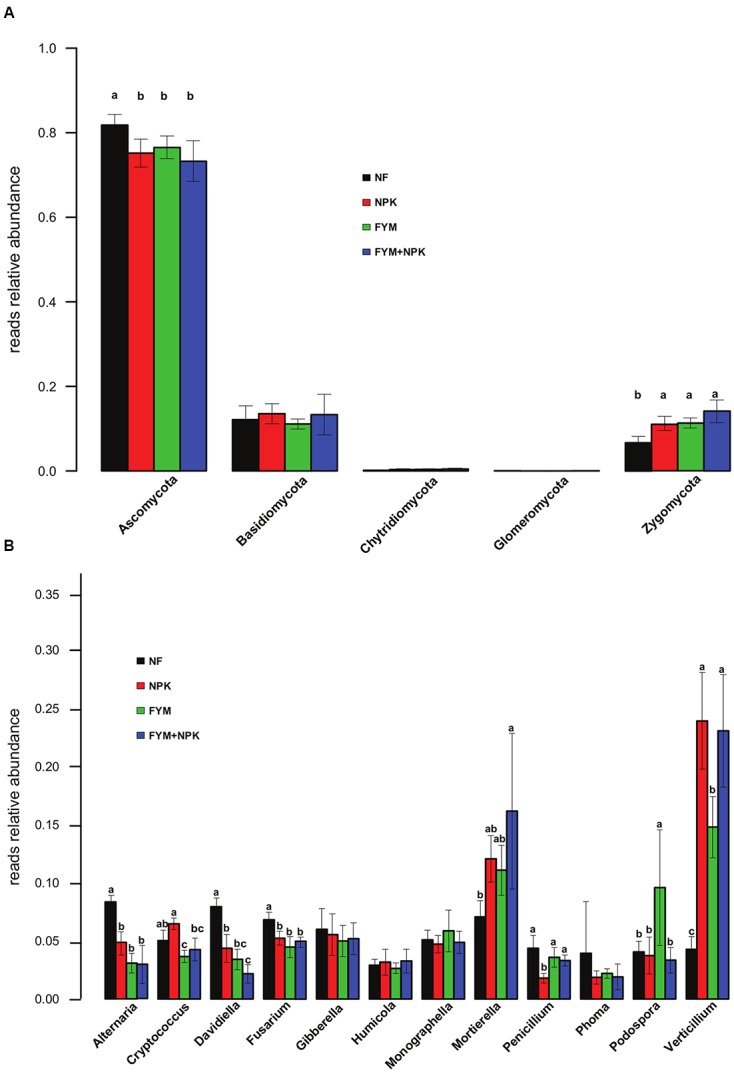
**Relative abundances of the major fungal phyla (A) and genera (B) in the four soils studied.** Different letters indicate significant differences based on Tukey’s HSD test *p* < 0.05. Error bars represent standard deviation (*n* = 5).

Long-term effects of fertilization on the fungal community were associated with shifts in the relative abundances of fungal taxa at the phylum level (**Figure 5A**). Compared to the unfertilized soil, long-term mineral and organic fertilization significantly increased the relative abundance of Zygomycota (*p* < 0.05). On the other hand, Ascomycota were significantly more abundant in the NF soil (*p* < 0.05) than in the fertilized ones. Within the Ascomycota, a significant increase in the relative abundance of Sordariomycetes and Tremellomycetes was observed in all fertilized treatments (NPK, FYM, FYM+NPK, *p* < 0.05; data not shown), while the NF soil showed a significantly higher proportion of Dothideomycetes and Leotiomycetes (*p* < 0.05; data not shown). No significant differences were detected in the relative abundances of the phyla Basidiomycota and Chytridimycota among the four soils studied. Only a few OTUs affiliated to the phylum Glomeromycota were recovered, probably due to limited coverage by the primers used. At the genus level, NF soil gathered significantly more reads assigned to the genera *Alternaria*, *Davidiella*, and *Fusarium* compared to the numbers from the fertilized soils (*p* < 0.05, **Figure 5B**). The opposite trend was observed for the genera *Mortierella* and *Verticillium*, which were more abundant in all fertilized soils (*p* < 0.05).

Analysis of the similarity revealed significant variation (*r* = 0.97, *p* < 0.001) among the four soils. Fitting the fungal taxonomic patterns against the NMDS axes revealed four distinct groups, each of which was representative of a specific fertilization regime (**Figure 4**). The PERMANOVA analysis confirmed these differences (**Table 2**): organic fertilization and mineral fertilization explained 19.4 and 22.8% of the dissimilarities in fungal community composition (*p* = 0.001 in both cases).

### Relationships between Microbial Community Composition and Soil Chemical Properties

The dbRDA model analysis identified distinct soil chemical properties which explained the changes in bacterial and fungal community structure. Soil pH (*F* = 8.795, *p* = 0.005) and TOC (*F* = 4.295, *p* = 0.005) were the edaphic properties having the strongest influence on the bacterial community structure (**Table 3**), including shifts in the dominant bacterial phyla. In contrast, fungal community composition was affected by nitrate (*F* = 2.780, *p* = 0.005), P_(DL)_ (*F* = 4.639, *p* = 0.005), and pH (*F* = 4.874, *p* = 0.005; **Table 4**). The NH_4_^+^-N and NO_3_^-^-N pools were the most influential factors within the phyla Ascomycota and Basidiomycota, which were also influenced in a smaller extent by TN and P_(DL)_, respectively.

**Table 3:** Relationships between the predictor soil variables and the bacterial community structure.

	pH		TOC	
	*F*	*p*	*F*	*p*
Bacteria	**8.795**	**0.005**	**4.295**	**0.005**
Acidobacteria	**6.739**	**0.005**	**2.649**	**0.005**
Actinobacteria	**13.345**	**0.005**	**6.090**	**0.005**
Bacteroidetes	**1.945**	**0.005**	1.518	0.095
Chloroflexi	**12.055**	**0.005**	**3.034**	**0.005**
Firmicutes	**1.930**	**0.010**	0.944	0.540
Gemmatimonadetes	**4.715**	**0.005**	**3.202**	**0.005**
Nitrospirae	**7.020**	**0.005**	**1.873**	**0.025**
Proteobacteria	**4.295**	**0.005**	**2.616**	**0.005**

*Results show marginal tests using the dbRDA model. Significant p-values less than 0.05 are indicated in bold*.

**Table 4:** Relationships between the predictor soil variables and the fungal community structure.

	pH		NO_3_^-^-N		NH_4_^+^-N		TN		P_(DL)_	
	*F*	*p*	*F*	*p*	*F*	*p*	*F*	*p*	*F*	*p*
Fungi	**4.874**	**0.005**	**2.780**	**0.005**	1.370	0.210	0.920	0.550	**4.639**	**0.005**
Ascomycota	1.460	0.260	**5.432**	**0.005**	**4.906**	**0.005**	**5.281**	**0.005**	0.922	0.520
Basidiomycota	0.995	0.420	**4.709**	**0.005**	**2.886**	**0.020**	0.593	0.710	**2.635**	**0.015**
Chytridimycota	0.662	0.650	0.721	0.610	0.615	0.720	0.294	0.940	0.234	0.930
Zygomycota	**10.113**	**0.005**	0.635	0.560	0.235	0.890	0.132	0.940	2.564	0.125

*Results show marginal tests using the dbRDA model. Significant p-values less than 0.05 are indicated in bold.*

## Discussion

### Fertilization Effects on Soil Edaphic Properties and Microbial Biomass

In this study, we analyzed soil samples from the “Static Fertilization Experiment Bad Lauchstädt” to survey the effect of different fertilization strategies, carried out for more than a century, on the edaphic properties and on the soil microbiota in agricultural soils. All the soil nutrients measured were higher in the manure-based treatments (FYM and FYM+NPK) compared to the unfertilized soil (**Table 1**), substantiating that applications of manure over a long period support the buildup of soil organic matter and thus improve soil fertility (Fließbach et al., 2007; Gattinger et al., 2012; Giacometti et al., 2014). A lower, but significant increase of TOC (11% on average) was also observed in the long-term mineral fertilized soil. Körschens et al. (2013) recently obtained a similar result analyzing data from other agricultural long-term experiments located in Europe. This study revealed that mineral NPK fertilization increased organic C by 10% on average compared to the unfertilized control plots. Substantially positive effects of mineral fertilization on soil nutrient and carbon pools are likely caused by increased crop biomass production and stabilization of organic carbon in the soil (Ludwig et al., 2011; Giacometti et al., 2013). TOC was strongly correlated with MBC, and a positive effect on microbial biomass by organic inputs has been well documented in long- (Murugan and Kumar, 2013; Stagnari et al., 2014; Frossard et al., 2016) and short-term (Lagomarsino et al., 2009; Lazcano et al., 2013; Ma et al., 2016) agricultural studies. In accordance with the TOC:MBC correlation, a slight increase in MBC was also found after long-term mineral fertilization, which is in line with a recently published meta-analysis of agricultural systems, where the authors concluded on a positive effect of inorganic fertilizer on microbial biomass over time (Geisseler and Scow, 2014). Crop biomass was strongly correlated with nutrient availability and was markedly higher in the fertilized treatments compared to the control soil (Supplementary Table S2), although we did not find any relation between plant biomass, MBC and microbial activity (Supplementary Figure S5). The highest crop yield was obtained in the treatments characterized by the application of mineral fertilizers, which showed a significant lower pH than the NF soil. The low soil pH detected in the NPK treatment was likely caused by the long-term input of ammonium-containing fertilizers, which enhance soil acidification (Fierer et al., 2007; Geisseler and Scow, 2014). Compared to the unfertilized soil, a slightly lower pH was also observed in the FYM fertilized soil. Animal manure contains N in reduced forms and their mineralization and oxidation produces H^+^ ions and acidity (Pandey et al., 2007). Furthermore, decomposition of animal manure may lead to the production of organic acids, which release H^+^ into the soil as well. On the other hand, application of manure can also counteract soil acidification, evident by comparing FYM+NPK and NPK soils, by introducing calcium carbonates and bicarbonates (resulting from cattle supplemental feed) into the soil (Li et al., 2012; Dong et al., 2014). Moreover, organic matter itself acts as a buffer through reversible protonation of organic acids and additionally reduces the concentration of aluminum in the soil solution by adsorption processes (Naramabuye and Haynes, 2006).

### Long-Term Fertilization Drives Soil Microbial Community Structure and Activity

Long-term organic and mineral fertilization had significant impacts on the soil microbial community structure and activity. NMDS ordination plots as well as PERMANOVA and ANOSIM analyses revealed clear shifts in bacterial and fungal community composition in relation to fertilization treatments (**Figure 4**; **Table 2**). Several studies have reported that both mineral and organic long-term fertilization led to changes in soil microbial community composition (Lentendu et al., 2014; Hartmann et al., 2015; Eo and Park, 2016), while significant changes in soil microbial community structure and activity in the short term are not always observed (Stark et al., 2007; Roberts et al., 2011; Geisseler and Scow, 2014), indicating a different response of the microbial community in relation to short- and long-term nutrient additions. In our study, the differences in microbial community composition were further mirrored by different proportions of fungal and bacterial groups found in the four soils surveyed (**Figures 3** and **5**). Proteobacteria and Firmicutes were significantly more abundant in the FYM treatments compared to NPK and NF soils. Members of both phyla have generally been described as copiotrophic bacteria (Fierer et al., 2007; Lienhard et al., 2014), which are fast-growing organisms that prefer carbon-rich environments. It is likely that long-term fertilization with manure, which contributes to there being high amounts of TOC and TN in the upper soil layers (Ludwig et al., 2011), promoted the growth of populations of these two phyla. Moreover, Proteobacteria and Firmicutes are common in fecal matter, and thus each application of farmyard manure would have added an external community to the soil; this may have contributed to the changes in soil community composition (Shanks et al., 2011). On the other hand, Acidobacteria were more abundant in the unfertilized soil compared to the fertilized ones and a negative correlation between this phylum and pH has been widely reported (Rousk et al., 2010; Fierer et al., 2012; Liu et al., 2015). Acidobacteria are generally classified as slow-growing oligotrophs (Fierer et al., 2007) and recent long- (Yao et al., 2014; Eo and Park, 2016) and short-term (Herzog et al., 2015) studies found that their abundance decreased significantly with N fertilizer application. Conversely, Actinobacteria, which represent the predominant phylum in all the soils studied and play a major role in agricultural soil quality promotion as they are remarkable organic matter decomposer (Strap, 2011), were significantly more abundant in the NPK treatment than in the other soils surveyed. This result may be explained by a negative correlation between the abundance of Actinobacteria and soil pH, recently described by Li et al. (2012).

In the fungal dataset, the majority of the fungal OTUs were assigned to the Ascomycota (**Figure 5A**), which is one of the most diverse and ubiquitous phyla of eukaryotes. They are important decomposer of organic substrates (e.g., wood, leaf litter, and dung) and a previous study have already reported Ascomycota as the predominant fungal phylum in agro-ecosystems (Lienhard et al., 2014). A low quantity of sequences related to members of the Basidiomycota was recovered from all soil samples surveyed. Recent studies have found that both, Basidiomycota and Ascomycota, represent the main soil fungal decomposers (Ma et al., 2013; Weber et al., 2013). However, while fungi from the basidiomycete phylum are particularly important in forests for degrading plant litter with high lignin content (Blackwood et al., 2007), their abundance in cropping systems may be lower. An increase in the recovery of Zygomycota sequences was also observed in all the fertilized treatments compared to the unfertilized soil. Members of the this phylum are mostly saprophytic and play an important role in the decomposition of plant debris and other more resistant materials (Richardson, 2009). It is likely that the increase in nutrient availability delivered by long-term fertilization have led to the increase of their abundance. In our study only few OTUs were detected as Glomeromycota. This is mainly due to the fact that in this study, we used primer pair targeting the ITS rDNA region and widely used for fungal community analysis across different ecosystems (Orgiazzi et al., 2012; Wubet et al., 2012; Tedersoo et al., 2014; Hartmann et al., 2015). Although the ITS region has been suggested as the standard fungal barcode (Schoch et al., 2012), the primers pairs bias for detection of Glomeromycota (Stockinger et al., 2010; Kohout et al., 2014) leading to an underestimation of this phylum in this study. However, since the soil sampling was carried out after the crop harvest and before sowing the new crops, the contribution of Glomeromycota on the overall microbial activity might be relatively small, as members of this phylum depend on the association with living plant roots to fulfill their life cycle (Antunes et al., 2012).

Overall, the bacterial community structures were driven by soil pH and TOC content (**Table 3**), while pH, available phosphorus and nitrogen pools accounted for the shifts in fungal communities structures observed among the treatments (**Table 4**). These findings were consistent with previous studies that indicated pH as the main factor that influenced microbial community composition in agro-ecosystems (Hartman et al., 2008; Lauber et al., 2008; Zhalnina et al., 2015). In line with recent studies (Lauber et al., 2009; Rousk et al., 2010; Joa et al., 2014), we found that soil pH, besides TOC, is the main predictors of microbial diversity (Supplementary Figure S5). A number of mechanisms may account for the association between pH and microbial community composition, as soil pH may select some microbial taxa over others (Rousk et al., 2010), besides affecting nutrient availability (Kemmitt et al., 2006).

Although we observed significant differences in the bacterial and fungal community structures among the four treatments, all the fertilized soils showed a higher microbial activity compared to the unfertilized one. The soil hydrolases studied, β-glucosidase, cellobiosidase, xylosidase, *N*-acetylglucosaminidase and phosphatases, catalyze reactions involved in the biogeochemical transformations of C, N, and P in the soil and are widely used for assessing soil microbial activity and substrate mineralization (Burns et al., 2013; Sinsabaugh et al., 2014). Enhanced microbial activity as a consequence of organic manure applications have already been reported in short- (Dinesh et al., 2010; Lazcano et al., 2013) and long-term experiment (Giacometti et al., 2014; Zhang et al., 2015). Higher hydrolase activities were also found in the NPK soil compared to NF and other recent studies have shown that inorganic fertilization can also promote the activity of cellulases (Geisseler and Scow, 2014; Jian et al., 2016), chitinase (Zhang et al., 2015) and phosphatases (Marklein and Houlton, 2012; Jian et al., 2016) in agricultural soils. However, interestingly, *N*-acetylglucosaminidase and phosphatase activities were higher in the mineral fertilized soils (NPK and FYM+NPK) compared to the organic fertilized soil indicating that long-term mineral fertilization considerably affects the enzymes involved in N and P cycling in soil. Application of N mineral fertilizers have been related to an increase in C and N mineralization due to changes in soil quality by long-term mineral fertilization (Mulvaney et al., 2009; Frossard et al., 2016). On the other hand, mineral fertilization (in particular when ammonia based fertilizers are used) prompts soil acidification, which in turn decreases P availability (Geisseler and Scow, 2014). The reduced P availability, along with N addition, raises the N/P ratio, increases P demand, and consequently may enhances the production and secretion of phosphatases (Marklein and Houlton, 2012; Frossard et al., 2016).

In order to link the obtained activities to the respective microbial communities we calculated the biomass-specific enzyme activities (enzyme activity related to the microbial biomass; **Table 5**). Landi et al. (2000) have suggested that this activity to biomass ratio provides more meaningful information on soil microbial activities and functions. Intriguingly, we found that the addition of FYM does not significantly increase the biomass-specific enzyme activities in comparison to the unfertilized soil. Conversely, for all the hydrolases measured with the exception of cellobiosidase, an increase in the biomass-specific activity with mineral fertilization was observed. Moreover, NPK soil showed particular high biomass-specific activities of *N*-acetylglucosaminidase and phosphatase. These findings indicated a more pronounced enzyme activity carried out by the microbial populations harbored in the NPK soil compared to the other treatments. Changes in enzyme production might either be physiological (Stone et al., 2014) or they might result from a shift in microbial community composition (Kaiser et al., 2014). In our study, we observed a distinct microbial community harbored in the NPK soil and, interestingly, we recovered a significantly higher proportion of Actinobacteria sequences in the NPK soil compared to the other treatments (**Figure 3**). Actinomycetes are among the most notable prokaryotic chitin degraders (Metcalfe et al., 2002; Hjort et al., 2010), and the high activity of *N*-acetylglucosaminidase, which participates in the processes whereby chitin is converted to amino sugars, may be related to the high abundance of Actinobacteria in the NPK soil.

**Table 5:** Means ± standard deviations for enzymatic activities per unit of microbial biomass C in the four soils studied.

	NF	NPK	FYM	NPK+FYM
β-Glucosidase	0.674 b ± 0.25	1.19 a ± 0.32	0.76 ab ± 0.03	0.73 ab ± 0.03
Cellobiohydrolase	0.041 b ± 0.01	0.15 a ± 0.06	0.07 ab ± 0.03	0.09 ab ± 0.03
*N*-Acetylglucosaminidase	0.103 b ± 0.04	0.32 a ± 0.07	0.14 b ± 0.04	0.14 b ± 0.03
Phosphatase	0.791 b ± 0.27	3.16 a ± 0.35	0.76 b ± 0.21	1.1 b ± 0.27
Xylosidase	0.076 a ± 0.03	0.14 a ± 0.04	0.09 a ± 0.02	0.09 a ± 0.03

*For each row different letters indicates that the mean value differs significantly among the soil studied (p < 0.05) based on Tukey’s HSD test.*

### Abundance of Agriculturally Relevant Microbes Are Driven by Fertilization Regime

When exploring the ecological data obtained by pyrosequencing, we looked for particular taxa known to be beneficial or pathogenic that are promoted or suppressed by different management strategies, although we can only speculate on the ecological role of the these detected taxa based on what has previously been described in other systems (Hartmann et al., 2015). It has been estimated that crop losses caused by plant diseases amount annually to around $220 billion worldwide (Agrios, 2005). In this light, knowledge on how fertilization strategies may enhance the proliferation of taxa that are beneficial, detrimental and/or pathogenic to crop plants represents invaluable information. For instance, *Verticillium* species, which are major plant pathogens that induce wilt diseases in a wide range of mainly dicotyledonous hosts, increased significantly in their relative abundance in soils treated with mineral fertilizer (NPK and NPK+FYM). Conversely, several potential plant pathogenic fungal genera affiliated to the phylum Ascomycota, *Fusarium*, *Phoma*, and *Alternaria* were more abundant in the unfertilized soil. Concerning the beneficial organisms, one of the most abundant fungal genera detected in this survey, *Mortierella*, which has been investigated as potential biological control agent against several plant pathogens (Tagawa et al., 2010) and recently as a means of insect pest-control (Edgington et al., 2014), was also positively associated with farmyard manure and mineral fertilization. Members affiliated to *Streptomyces*, a well-studied genus of saprophytic Actinomycetes with vast economic importance in agricultural systems because they include many potential biocontrol agents (Schrey and Tarkka, 2008) but also some plant pathogens were more abundant in all fertilized soils compared to the unfertilized soil. These observations demonstrated that particular fertilization treatments can select, promote or reduce specific groups of beneficial or detrimental soil microorganisms. Further research should be addressed to increase our understanding of the impacts of specific management strategies on soil microorganisms and soil microbial processes in order to increase crop yields under sustainable agricultural production systems.

## Conclusion

The multidisciplinary approach presented in this study allowed us to carry out an in-depth survey of the effects of long-term fertilization on the activity and composition of soil microbial communities. Fertilization with organic manure, which has a more diverse composition in terms of macro- and micro-nutrients than the mineral fertilizers, was responsible for the strong enhancement in soil microbial biomass and diversity. In contrast, long-term application of inorganic fertilizer does only slightly increase biomass but strongly enhances biomass-specific enzyme activities in the soil. Moreover, particular fertilization treatments could select, promote or reduce specific groups of beneficial or detrimental soil microorganisms and our results give indications that organic fertilization supports some beneficial, while suppressing detrimental taxa. Studies that compare to productivity and stability of differently fertilized agricultural systems indicated that the application of farmyard manure in combination with mineral fertilizer usually result in high crop yield. According to our results this outcome may not only based on the increased soil nutrient contents *per se* but also to beneficial changes in the soil microbial community, which include higher diversity and an enrichment of beneficial microorganisms, brought about by manure application and an enhanced microbial activity induced by mineral fertilization.

## Author Contributions

DF, FB, and TR planned the study. DF and TR collected the samples. DF and TR performed the lab work. ES contributed to the physiochemical data of soil samples. DF, ES, GL, and TW analyzed the data and provided general guidance. DF wrote the manuscript. GL, ES, FB, TW, and TR contributed in reviewing the manuscript.

## Conflict of Interest Statement

The authors declare that the research was conducted in the absence of any commercial or financial relationships that could be construed as a potential conflict of interest.
