# Supplementary material for: Mineral vs. Organic Amendments: Microbial Community Structure, Activity and Abundance of Agriculturally Relevant Microbes Are Driven by Long-Term Fertilization Strategies
Source: Front Microbiol. 2016 Sep 14;7:1446. doi: 10.3389/fmicb.2016.01446 (PMC5022044; doi:10.3389/fmicb.2016.01446)
Supplement: Supplementary file 1 [file Data_Sheet_1.DOCX]

Supplementary Material

**Mineral vs. organic amendments: microbial community structure, activity and abundance of agriculturally relevant microbes are driven by long-term fertilization strategies**

**Authors**: Davide Francioli*****, Elke Schulz, Guillaume Lentendu, Tesfaye Wubet, François Buscot, Thomas Reitz

*** Correspondence:** Davide Francioli, Helmholtz Centre for Environmental Research - UFZ, Department of Soil Ecology, Theodor-Lieser-Str. 4, Halle (Saale), 06120, Germany

Email address: [davide.francioli@ufz.de](mailto:davide.francioli@ufz.de)

# Supplementary Figures and Tables

## Supplementary Figures

**Supplementary Figure 1.** “Static Fertilization Experiment” design and soil sampling strategy. The upper part of the figure represents the “Static Fertilization Experiment” near Bad Lauchstädt and the red squares indicate the plots sampled for this study. The lower part of the figure illustrates the soil sampling strategy.


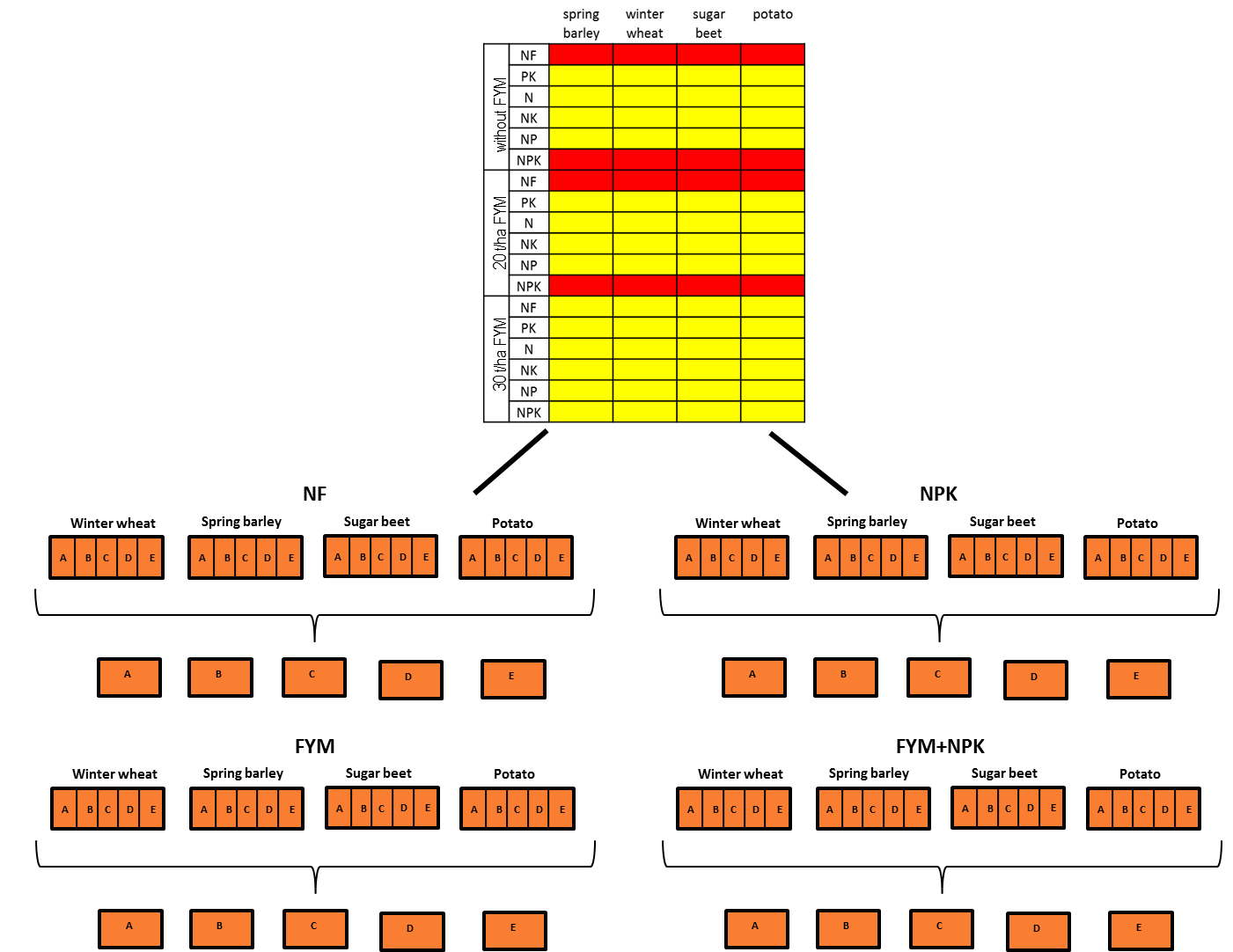


**Supplementary Figure 2.** Rarefaction curves for the **(A)** bacterial and **(B)** fungal dataset calculated at the minimal sequencing depth among samples belonging to the same datasets after the removal of singletons, doubletons and tripletons OTUs.


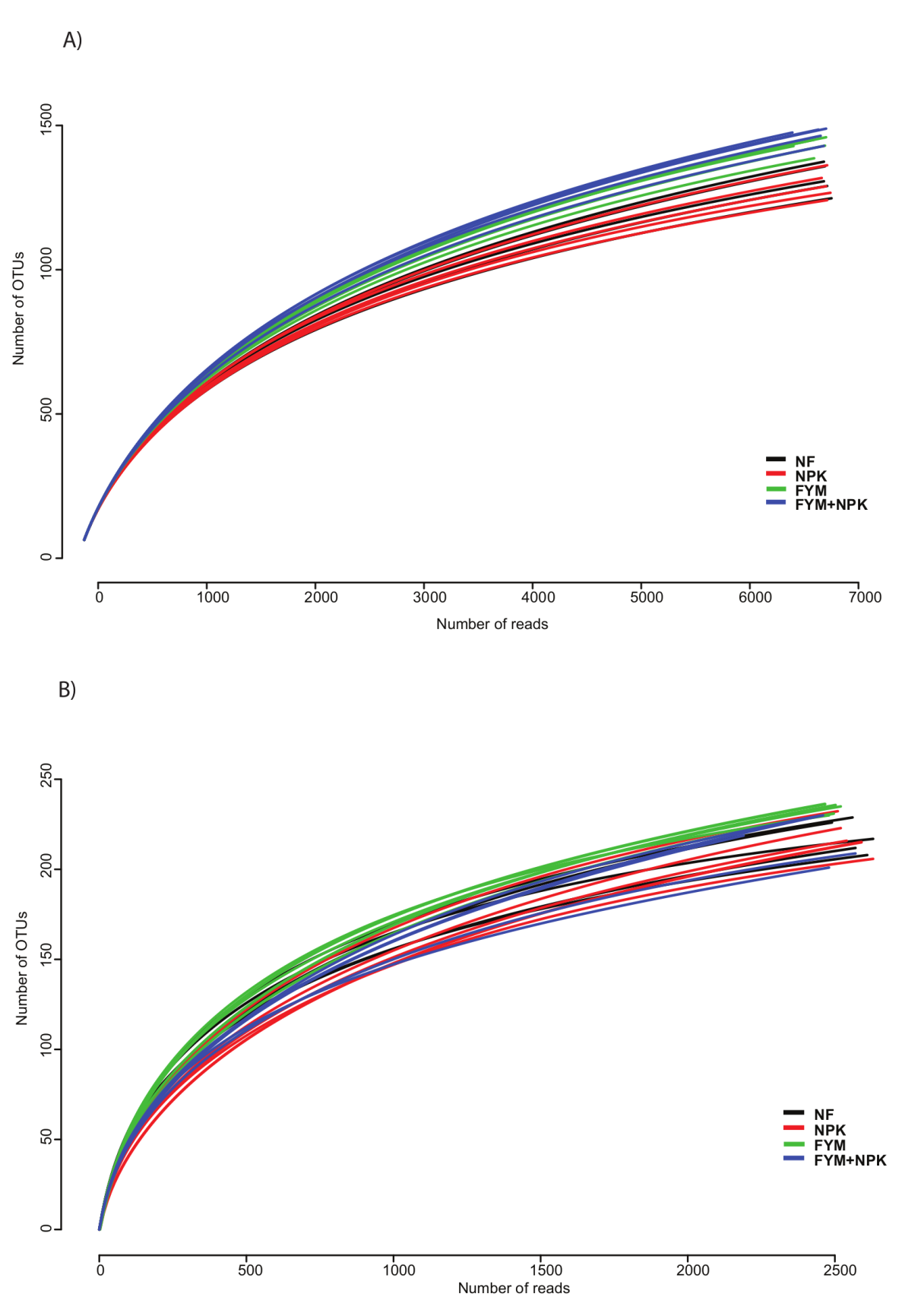


**Supplementary Figure 3.** Relative abundances of the phyla detected within the bacterial dataset.


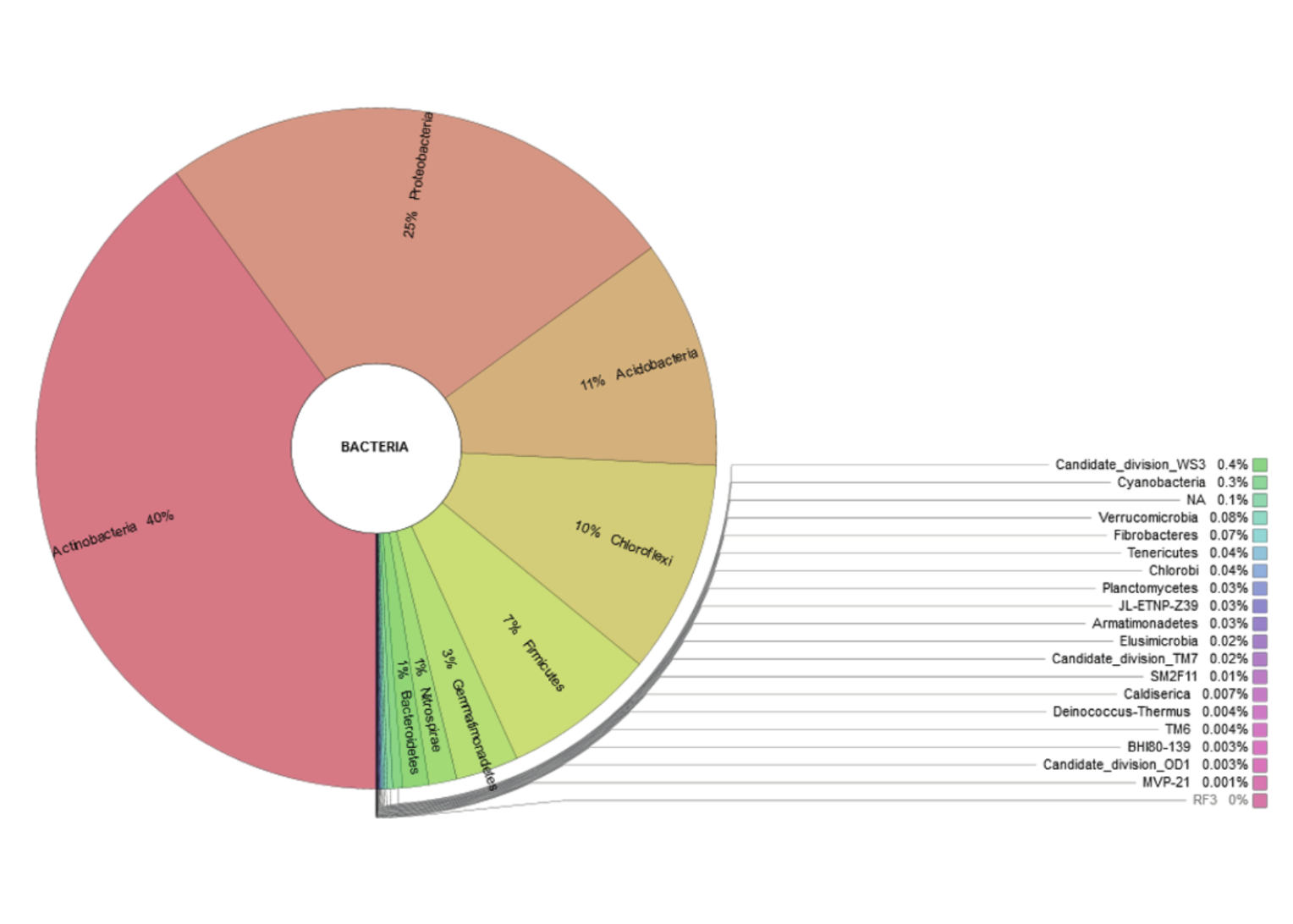


**Supplementary Figure 4.** Relative abundances of the phyla detected within the fungal dataset.


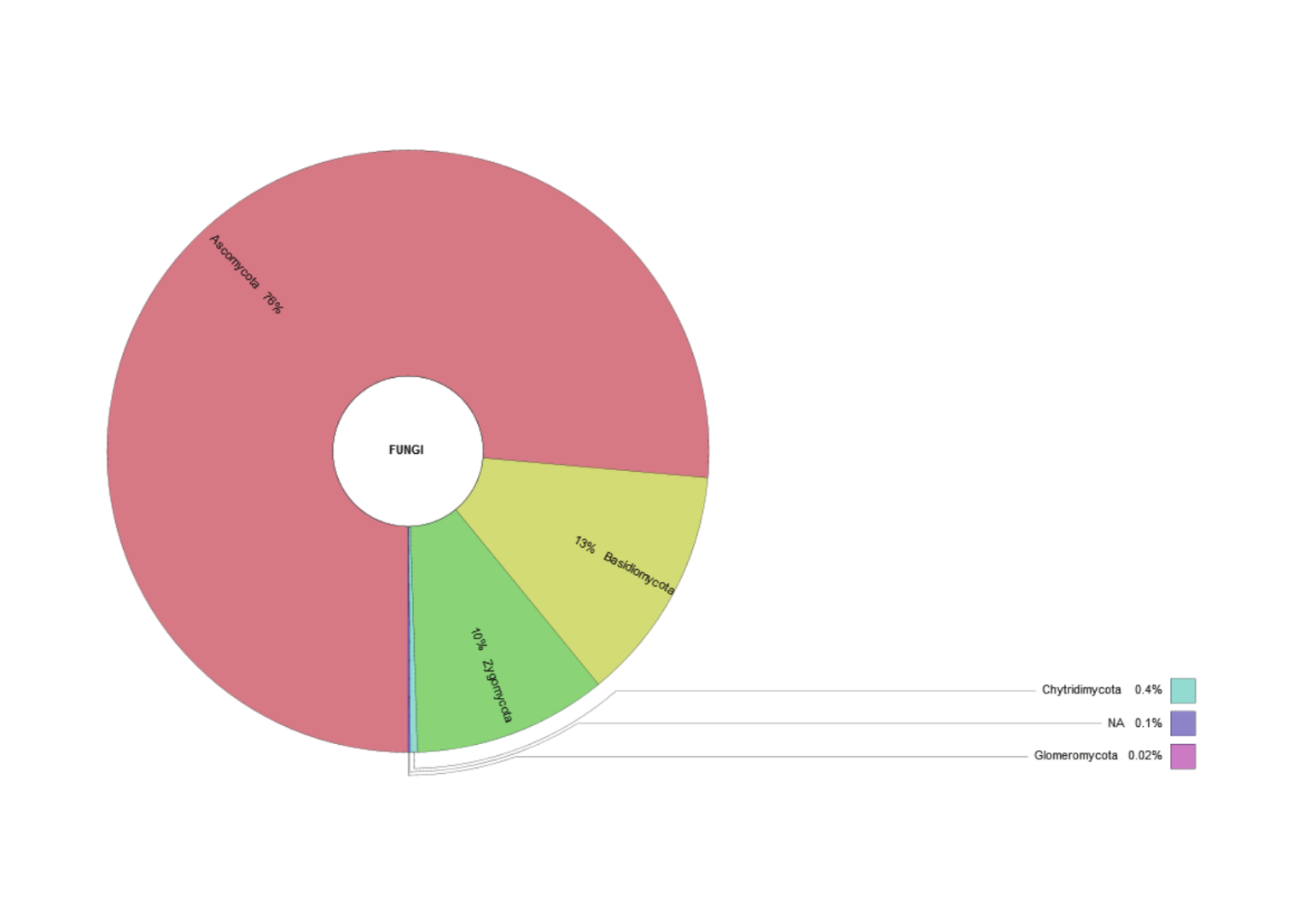


**Supplementary Figure 5**

Final path models of soil edaphic properties as predictors of plant biomass, soil diversity and enzyme activity. TOC and TN were strongly correlated (r= 0.97; p<0.001) therefore, we run separate analyses with each variable; (a) with TOC and (b) with TN. Solid black arrows represent significant positive paths (P<0.05) derived from SEM analysis, solid red arrows represent significant negative paths (P <0.05) and dotted arrows represent retained but non-significant paths (P >0.05). Standardized path coefficients are reported next to the arrows. Overall fit of piecewise SEMs was evaluated using Fisher’s C statistic (if P>0.05, derived from a Chi-squared distribution, indicates good model fit) and Akaike information criterion (AIC; lower AIC values indicate better fit). PBM, plant biomass; MDV, microbial diversity; MBC, microbial biomass carbon; BG, β-glucosidase; XYL, Xylosidase; NAG, N-acetylglucosaminidase; CB, Cellobiohydrolase; PHOS, Phosphatase. A soil biodiversity index (MDV) was calculated from the average of all standardized soil community characteristics which included bacterial and fungal richness.

**
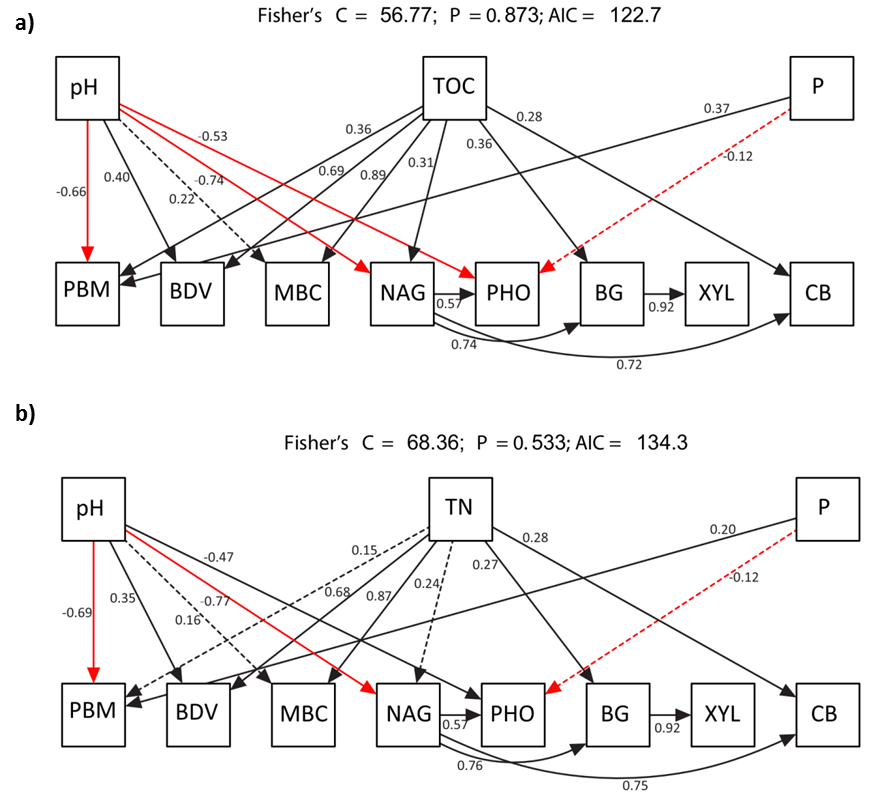
**

## Supplementary Tables

| **Table S1.** Fertilization treatments in the “Static Fertilization Experiment”. | | | | | |  |
| --- | --- | --- | --- | --- | --- | --- |
| **Treatment** | FYM  t * ha^-1^ * 2y^-1^ | N kg * ha^-1^  ZB SB PO WW | P kg * ha^-1^  ZB SB PO WW | | K kg * ha^-1^  ZB SB PO WW |  |
| NF | 0 | 0 0 0 0 | 0 0 0 0 |  | 0 0 0 0 |  |
| NPK | 0 | 170 80 140 100 | 60 0 60 0 |  | 230 0 230 0 |  |
| FYM | 20 | 0 0 0 0 | 0 0 0 0 |  | 0 0 0 0 |  |
| FYM+NPK | 20 | 150 60 120 80 | 28 0 28 0 |  | 110 0 110 0 |  |

FYM: Farmyard manure; N: nitrogen; P: phosphorus; K: potassium; ZB: sugar beet; SB: spring barley; PO: potatoes; WW: winter wheat.

|  | **Table S2.** Crop biomass harvested in 2012 in the treatments investigated.  In parenthesis are reported the relative yield of each crop related to the unfertilized control. | | | | | | | | | | | | | | | | |  |  |
| --- | --- | --- | --- | --- | --- | --- | --- | --- | --- | --- | --- | --- | --- | --- | --- | --- | --- | --- | --- |
|  | | Spring barley | | Winter wheat | | |  | | | Potatoes | Sugar beet | | | Mean rel. yield^a^ | | |  |  |  |
|  | | t ha^-1^ dry matter | | |  | | | | t ha^-1^ fresh matter | | | |  | | |  |  |  |  |
| NF | | 23 (1) | 46 (1) | | |  | | 166 (1) | | | | 445 (1) | | | 1 | | | |  |
| NPK | | 83 (3.57) | 98 (2.12) | | |  | | 243 (1.46) | | | | 685 (1.53) | | | 2.17 | | | |  |
| FYM | | 61 (2.63) | 70 (1.52) | | |  | | 292 (1.75) | | | | 546 (1.22) | | | 1.78 | | | |  |
| FYM+NPK | | 84 (3.63) | 101 (2.18) | | |  | | 221 (1.32) | | | | 674 (1.51) | | | 2.16 | | | |  |

^a^Mean rel.yield, mean of the relative crop yield in each treatment.

| **Table S3**  **S**oil enzymes assayed for potential activity. | | |  | |  |
| --- | --- | --- | --- | --- | --- |
| Enzyme | EC | Substrate | | Concentration  in the assay | |
| β-glucosidase | EC 3.2.1.21 | 4-MUB-β-D-glucoside | | 200 µM | |
| Cellobiohydrolase | EC 3.2.1.91 | 4-MUB-β-D-cellobioside | | 150 µM | |
| N-acetylglucosaminidase | EC 3.2.1.14 | 4-MUB-*N*-acetyl-β-D-glucosaminide | | 150 µM | |
| Xylosidase | EC 3.2.1.37 | 4-MUB-β-D-xyloside | | 300 µM | |
| Phosphatase | EC 3.1.3 | 4-MUB-phosphate | | 200 µM | |
| EC, enzyme commission classification; MUB, methylumbelliferyl. | | |  | |  |

| **Table S4.**  Bacterial and fungal observed and estimated richness in each soil sample analyzed in this study. Numbers behind the dash indicate the replicate within a treatment. | | | | | | | |  |
| --- | --- | --- | --- | --- | --- | --- | --- | --- |
| **Bacteria Fungi** | | | | | | | |  |
|  | Sobs^a^ | Chao1^b^ | % Inv. Comp.^c^ |  | Sobs^a^ | Chao1^b^ | % Inv. Comp.^c^ |  |
| NF-1 | 1184 | 1655 | 72 |  | 217 | 246 | 88 |  |
| NF-2 | 1295 | 1839 | 70 |  | 208 | 270 | 77 |  |
| NF-3 | 1243 | 1690 | 74 |  | 226 | 273 | 83 |  |
| NF-4 | 1227 | 1680 | 73 |  | 212 | 277 | 76 |  |
| NF-5 | 1311 | 1824 | 72 |  | 229 | 301 | 76 |  |
| NPK-1 | 1203 | 1564 | 77 |  | 216 | 278 | 78 |  |
| NPK-2 | 1226 | 1729 | 71 |  | 206 | 244 | 84 |  |
| NPK-3 | 1178 | 1602 | 74 |  | 223 | 289 | 77 |  |
| NPK-4 | 1255 | 1759 | 71 |  | 239 | 301 | 79 |  |
| NPK-5 | 1299 | 1825 | 71 |  | 202 | 282 | 72 |  |
| FYM-1 | 1396 | 2018 | 69 |  | 228 | 285 | 80 |  |
| FYM-2 | 1367 | 1952 | 70 |  | 214 | 286 | 75 |  |
| FYM-3 | 1323 | 1727 | 77 |  | 231 | 280 | 83 |  |
| FYM-4 | 1365 | 1847 | 74 |  | 235 | 297 | 79 |  |
| FYM-5 | 1387 | 1929 | 72 |  | 227 | 305 | 75 |  |
| FYM+NPK-1 | 1422 | 2044 | 70 |  | 221 | 311 | 71 |  |
| FYM+NPK-2 | 1412 | 1983 | 71 |  | 230 | 314 | 73 |  |
| FYM+NPK-3 | 1366 | 1911 | 71 |  | 209 | 245 | 85 |  |
| FYM+NPK-4 | 1401 | 2057 | 68 |  | 211 | 286 | 70 |  |
| FYM+NPK-5 | 1426 | 2004 | 71 |  | 228 | 325 | 70 |  |
| ^a^S_obs_, Observed richness  ^b^Chao1, Estimate of OTU richness  ^c^Total observed richness/Chao1 estimate ×100. | | | | | | | |  |
